# A Novel Framework for Optimizing Peri-Implant Soft Tissue in Subcrestally Placed Implants in Single Molar Cases: Integrating Transitional and Subcrestal Zones for Biological Stability

**DOI:** 10.3390/jcm14072435

**Published:** 2025-04-03

**Authors:** Chiyun Won

**Affiliations:** Private Clinic, Chiyun Won Dental Clinic 2F, 46-1 Changjeon-ro, Mapo-gu, Seoul 04087, Republic of Korea; wonchiyun@gmail.com

**Keywords:** peri-implant soft tissue, subcrestally placed implants, emergence profile, biologic width, supracrestal tissue attachment, transitional zone, subcrestal zone, self-sustained soft tissue, peri-implant health, three-dimensional analysis, CBCT

## Abstract

**Background/Objectives:** The peri-implant soft tissue seal is crucial for the long-term success of subcrestally placed implants (SPIs). However, conventional biologic width—now referred to as supracrestal tissue attachment (STA)—models, originally developed for natural teeth, fail to account for the three-dimensional nature of peri-implant soft tissue adaptation. This study introduces a novel framework integrating the concepts of the transitional zone (TZ) and subcrestal zone (SZ) to systematically optimize peri-implant soft tissue architecture. **Methods:** A mathematical model was developed to determine the optimal implant placement depth by incorporating the emergence angle (EA), soft tissue thickness (STT), and peripheral crestal offset (PCO). Additionally, a three-dimensional peri-implant soft tissue analysis (3DSTA) approach utilizing cone beam computed tomography (CBCT) imaging was implemented to evaluate peri-implant soft tissue adaptation and emergence profile design. Clinical parameters were analyzed to establish guidelines for optimizing SPI placement depth and peri-implant soft tissue stability. **Results:** This study introduces the concept of self-sustained soft tissue (SSST), a biologically functional structure composed of the TZ and SZ, which enhances peri-implant health and stability. The proposed framework provides clinical guidelines for optimizing SPI placement depth, emergence profile contouring, and peri-implant soft tissue thickness to mitigate the risk of peri-implant mucositis. By shifting from a traditional two-dimensional perspective to a multidimensional analysis, this approach offers an evidence-based foundation for achieving biologically stable and esthetically predictable outcomes. **Conclusions:** The proposed three-dimensional model advances the understanding of peri-implant soft tissue adaptation by integrating novel anatomical and biomechanical concepts. By redefining peri-implant biologic width through the introduction of TZ and SZ, this study provides a structured framework for optimizing SPI placement and soft tissue management. Future research should focus on validating this model through histological studies and long-term clinical trials to refine its application in clinical practice.

## 1. Introduction

The evolution of implantology has transitioned from a bone-centric approach toward a more comprehensive focus on peri-implant soft tissue, recognizing its pivotal role in long-term implant stability. While early implant research primarily emphasized bone integration, contemporary studies have shifted their focus to peri-implant soft tissue, given its crucial influence on biological stability and long-term bone level maintenance. A well-structured peri-implant soft tissue seal not only contributes to long-term implant survival but also plays a fundamental role in protecting the underlying bone from resorption.

Beyond biological stability, the esthetic demands of implant dentistry have also increased, even in posterior restorations. Achieving natural soft tissue contours around implants is now a priority, leading to an increased emphasis on implant positioning and emergence profile design. To meet these demands, subcrestally placed implants (SPIs) have gained widespread adoption among clinicians. However, the increasing use of SPIs has also highlighted a critical gap in understanding—particularly regarding the three-dimensional peri-implant soft tissue adaptation in subcrestal implant placement.

Subcrestally placed implants (SPIs) have emerged as a versatile technique designed to address specific clinical challenges, including enhanced primary stability, improved esthetic outcomes, and better bone preservation compared to equicrestally placed implants. However, key concerns remain—particularly due to the lack of a well-defined model that explains the biologic width around implants in a three-dimensional context. This gap in understanding is especially evident when differentiating between pathological pockets, which indicate peri-implant disease, and the extended peri-implant soft tissue interface found around implant restorations. Despite these challenges, SPIs continue to gain traction as a technique aimed at achieving biologically stable and esthetically favorable outcomes [1,2,3,4].

The peri-implant soft tissue seal plays a critical role in the long-term success of SPIs. Unlike natural teeth, which have a well-defined supracrestal tissue attachment, peri-implant soft tissue must adapt dynamically to the implant–abutment complex to maintain biological stability. Conventional biologic width models, initially developed for natural dentition, have often been extrapolated to implants, despite fundamental structural and functional differences. However, these models fail to fully capture the three-dimensional complexity of peri-implant soft tissue adaptation, particularly in SPI cases where the implant is placed below the crestal bone level.

Several studies have emphasized the importance of soft tissue thickness, emergence profile design, and implant–abutment interface in peri-implant health. However, an integrated framework for optimizing peri-implant soft tissue stability, specifically for SPIs, remains underexplored. Existing classifications largely focus on bone-level implants without distinguishing the unique peri-implant tissue adaptations seen in SPI cases. Moreover, while studies have correlated peri-implant mucosal health with implant placement depth and emergence angle (EA), no structured model has been developed to quantify these relationships in a clinically applicable manner.

This study introduces a novel framework that integrates the concepts of the transitional zone (TZ) and subcrestal zone (SZ) to provide a more comprehensive understanding of peri-implant soft tissue adaptation. By incorporating a three-dimensional peri-implant soft tissue analysis (3DSTA) based on CBCT imaging and clinical evaluation, this study defines key parameters such as crest to restoration distance (CRD), soft tissue thickness (STT), and self-sustained soft tissue (SSST) to evaluate peri-implant soft tissue stability. Additionally, a mathematical model was developed to determine optimal SPI placement depth, taking into account emergence angle (EA), soft tissue thickness (STT), and peripheral crestal offset (PCO) to enhance peri-implant biological integration.

A novel diagnostic tool, Implant Paper Point Probing (IPPP), is also introduced in this study to assess peri-implant soft tissue sealing. This method allows for direct sulcus assessment, offering a simple yet effective means of distinguishing between healthy and compromised peri-implant soft tissue.

This study aims to bridge the gap between conventional biologic width (or STA) models and real-world peri-implant soft tissue dynamics by shifting from a two-dimensional STA concept to a three-dimensional analytical model. The findings provide new clinical guidelines for optimizing implant emergence profiles, determining SPI placement depth, and improving peri-implant soft tissue management. Future studies should validate this framework through histological investigations and long-term clinical trials to further refine peri-implant soft tissue optimization strategies.

## 2. Optimizing Emergence Profile and Placement Depth in Molar Implant Restorations

A fundamental principle in implant therapy is the recreation of natural tooth anatomy and function. Beyond mere replacement, successful implant restoration demands careful consideration of emergence profile design, particularly in the posterior region, where esthetics and function must coexist. Although the anterior region typically receives more attention regarding esthetic outcomes, molar restorations require equal attention to ensure harmonious soft tissue adaptation and biomechanical stability.

The emergence profile of the implant must facilitate a seamless transition between the restoration and surrounding tissues, mimicking the contours of a natural tooth. This involves meticulous planning of the submucosal portion, including the implant abutment and the crown’s cervical region, to ensure biologically stable and esthetically natural results.

### 2.1. Coronal Flaring: A Crucial Consideration in IPS Implants

In internal platform switching (IPS) implants, coronal flaring plays a critical role in achieving both biological stability and esthetic integration. Coronal flaring refers to the gradual widening of the implant restoration’s emergence profile, as it transitions from the implant fixture–abutment connection (FAC) to the cervical contour of the crown. This design ensures a natural emergence profile that blends seamlessly with the surrounding soft tissue.

However, there is a clinical debate regarding the ideal placement of coronal flaring relative to the peri-implant mucosa:

(a) Supramucosal coronal flaring: When flaring occurs above the mucosal level, the emergence profile remains entirely supramucosal. This results in a less natural transition between the implant restoration and peri-implant soft tissue, which may compromise esthetics, especially in visible regions.
(b) Submucosal coronal flaring: When flaring occurs beneath the mucosa, it allows for a progressive transition between the abutment and the soft tissue, leading to a more natural-looking emergence profile and improved esthetic outcomes.

### 2.2. Clinical Concerns About Submucosal Coronal Flaring

Some clinicians prefer supramucosal coronal flaring over submucosal flaring due to concerns about the potential formation of a deep peri-implant pocket, which could increase the risk of bacterial colonization and peri-implant disease. Their argument is based on the assumption that a longer peri-implant soft tissue interface may be more susceptible to pathogenic invasion.

However, this assumption is flawed. Submucosal coronal flaring is not only esthetically superior but also biologically advantageous. The elongated soft tissue seal, rather than being a source of vulnerability, reinforces peri-implant defense mechanisms by increasing the area of connective tissue attachment and vascular supply. This enhanced biologic width (or STA) around IPS implants provides greater resistance to microbial penetration and peri-implant disease, ensuring long-term stability.

### 2.3. Rationale for Supporting Submucosal Coronal Flaring

**Figure 1:**
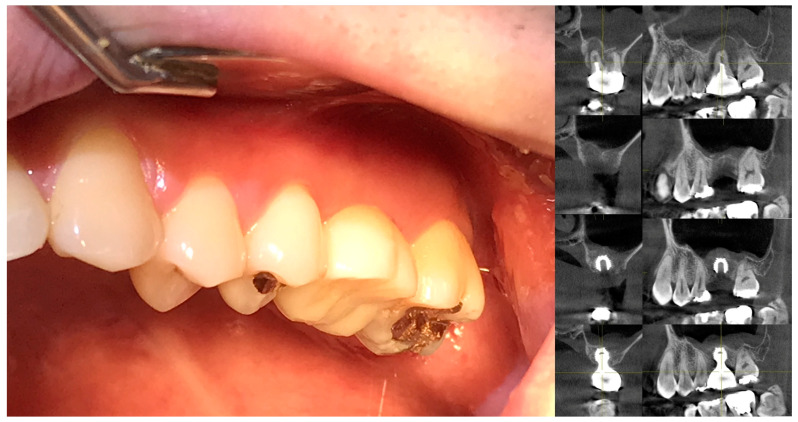
The upper first molar was replaced with an implant-supported restoration following the extraction of the natural tooth due to a periapical abscess. The replacement successfully restored both function and esthetics. Notably, attention should be given to the junction between the tooth structure (white) and the soft tissue (pink) in terms of their spatial relationship and proportional dimensions. Although the emerging part of the implant restoration is not externally visible, for a natural appearance, the emergence profile beneath the peri-implant soft tissue must align precisely with the same location and diameter as the cervical area of the neighboring natural teeth. This ensures that the implant restoration maintains continuity with the marginal gingiva at the soft tissue level and the cervical region of the adjacent natural teeth, achieving seamless integration within the oral environment.

Figure 1 highlights the importance of a carefully designed emergence profile in achieving a natural and biologically stable peri-implant soft tissue interface. In SPI cases, ensuring seamless soft tissue adaptation to implant restoration is critical. By placing coronal flaring submucosally, the transition between the implant and the soft tissue more closely mimics that of natural dentition, enhancing both esthetic and functional outcomes. However, this approach inevitably results in an extended and broader transitional zone within the peri-implant soft tissue.

The primary objective of peri-implant soft tissue management is to establish a biologically stable and esthetically harmonious integration between the implant and the surrounding gingiva. Increasing the contact area between the peri-implant mucosa and the implant restoration enhances soft tissue adaptation, leading to a more natural-looking appearance. However, this technique and its clinical applications require validation to confirm that an enlarged contact area not only increases biologic width but also reinforces the peri-implant soft tissue barrier against microbial infiltration.

To dispel the misconception that submucosal coronal flaring predisposes implants to peri-implantitis, it is essential to understand the broader contact zone of peri-implant soft tissue established in SPIs. The key question is whether this configuration results in a hazardous pocket prone to microbial infiltration or a biologically protective seal that reinforces peri-implant health.

By integrating the transitional zone (TZ) and subcrestal zone (SZ) as components of self-sustained soft tissue (SSST), this study introduces a new framework for interpreting the broad contact area generated in SPIs. Grounded in both clinical observation and theoretical analysis, this model provides a more comprehensive understanding of peri-implant soft tissue behavior in subcrestally placed implants.

## 3. Biologically Stable and Esthetic Molar Implant Restoration (BEMIR)

For an implant restoration to achieve a natural appearance, the peri-implant soft tissue (pink) and the cervical portion of the implant restoration (white) must be proportional in size and aligned harmoniously with the gingival levels of adjacent teeth. As long as the surrounding gingiva remains healthy, ensuring this precise correspondence from the very first visible point of the restoration supports seamless and natural-looking integration within the oral environment (Figure 1).

To achieve this outcome, the submucosal portion of the implant restoration—including the abutment and cervical region of the crown—must be designed in accordance with morphological and biological principles. This ensures a progressive emergence profile, facilitates soft tissue adaptation, and enhances both esthetics and biological stability. The emergence profile must be meticulously planned to maintain a smooth transition between the implant restoration and peri-implant soft tissue, preventing abrupt contour changes that may compromise integration.

### 3.1. The Impact of Internal Platform Switching (IPS) on Emergence Profile Design

Internal platform switching (IPS) implants are widely preferred due to their advantages in marginal bone preservation, soft tissue stability, and reduced microleakage at the implant–abutment junction. However, their narrower fixture–abutment connection (FAC) compared to the supramucosal crown portion necessitates careful consideration in emergence profile design.

If not properly managed, this diameter discrepancy may lead to

- Inadequate soft tissue support, affecting peri-implant stability.
- Excessive horizontal contour discrepancies, disrupting the transition between the restoration and gingiva.
- Compromised esthetics, particularly if the coronal flaring is not optimally positioned relative to the peri-implant mucosa.

To mitigate these risks, submucosal contour planning—including strategic abutment selection and precise emergence profile design—is crucial. By properly shaping the transition from the FAC to the cervical contour of the crown, clinicians can ensure both biological stability and esthetic harmony.

### 3.2. Determining the Vertical Dimension for Optimal Emergence Profile

A key step in BEMIR planning is defining the required vertical distance (V) from the peri-implant soft tissue to the implant restoration’s starting point, i.e., the fixture–abutment connection (FAC). This is influenced by

- Horizontal emergence profile dimensions (difference between the FAC diameter and the cervical diameter of the crown).
- The emergence angle (EA) or flaring angle (FA), dictating the contour transition.

A refined understanding of coronal flaring mechanics—particularly the distinction between supramucosal and submucosal flaring—is essential in optimizing soft tissue adaptation and achieving a functional, esthetic emergence profile.

Table 1 presents the calculated vertical distances based on various emergence angles (EA) and horizontal differences, providing a guideline for bone-level implants with IPS designs.

It has been long since the emergence angle (EA) is more known in academic literature with its definition (angle formed between the long axis of implant and the lateral surface or the implant restoration). But clinically, using the flaring angle (FA) is more convenient. The FA is defined as the angle between the lateral surface of the implant restoration and the horizontal plane, which is perpendicular to the long axis of the implant) Simply, EA = 90 − FA. The FA is more nuanced when addressing the coronal flaring phenomenon, which is indispensable for IPS implants.

### 3.3. Flaring Angle (FA) vs. Emergence Angle (EA): A Practical Perspective

The emergence angle (EA) has long been the standard parameter in academic literature for describing the transition between an implant and its restoration. It is defined as the angle formed between the implant’s long axis and the lateral surface of the implant restoration. This definition has been widely accepted in theoretical discussions and research studies. However, in clinical practice, the flaring angle (FA) offers a more intuitive and practical alternative for evaluating the coronal flaring phenomenon, which is crucial for internal platform switching (IPS) implants.

### 3.4. Defining the Flaring Angle (FA)

The FA is measured as the angle between the lateral surface of the implant restoration and the horizontal plane, which is perpendicular to the implant’s long axis. This makes the FA more relevant for clinical applications, particularly when adjusting submucosal contours and ensuring optimal emergence profile design. Since the FA is measured from the horizontal reference, while the EA is measured from the implant’s long axis, they are related by the equation

EA = 90 − FA

This relationship highlights how the FA provides a direct representation of the horizontal flaring component, whereas the EA focuses on the vertical emergence transition.

While the EA remains essential in theoretical discussions, the FA provides a more clinically relevant and practical alternative—particularly for IPS implants, where the coronal flaring effect plays a critical role in soft tissue adaptation and implant esthetics.

## 4. Mathematical Approach to Determining Vertical Distance for Emergence Profile Optimization

In designing a biologically stable and esthetic molar implant restoration (BEMIR), a key factor is determining the necessary vertical distance (v) from the first visible point of the implant restoration above the peri-implant soft tissue to the fixture–abutment connection (FAC). This measurement is typically taken from the buccal first point (BFP)—the most coronal visible part of the implant restoration above the mucosa—and is based on the emergence angle (EA) or flaring angle (FA), where

FA = 90 − EA

This distance (v) is dependent on two factors:

1. The horizontal distance difference (d) between the cervical diameter of the restoration and the FAC diameter.
2. The emergence angle (EA), which describes the inclination of the lateral contour of the implant restoration relative to the implant axis.

To achieve a natural-looking, esthetic emergence profile, the diameter of the implant restoration at the BFP should correspond to the height of the contour of the designed restoration.

### 4.1. Mathematical Formula for Vertical Distance (v) Calculation

Assuming a flat edentulous crestal bone table (without accounting for peripheral crestal offset, PCO), the estimated vertical distance v can be determined using a fundamental trigonometric principle:

Tan (90 − EA) = v/d

Since

Tan (90 − EA) = cot (EA) = tan (FA)

we derive

v = d × cot (EA) = d/tan (EA) = d × tan (FA)

This formula allows clinicians to precisely calculate v, ensuring a smooth submucosal transition between the implant and peri-implant soft tissue. In subcrestally placed implants (SPIs), the proper determination of both vertical (v) and horizontal (d) distances is crucial for achieving a harmonious soft tissue interface and preventing excessive pressure on the peri-implant mucosa.

**Key Considerations:**

- The reference point for measuring v is at the center of the edentulous crestal surface (CP).
- v includes mucosal thickness, which typically varies between 2–3 mm.
- This calculation assumes a flat, edentulous ridge—a condition that is rare in clinical cases, necessitating adjustments for PCO.

### 4.2. Accounting for Peripheral Crestal Offset (PCO) in Total Vertical Distance Calculation

Before determining the actual depth of implant placement, it is necessary to account for the natural curvature of the edentulous ridge, as it is rarely flat.

- CP (Center Point): The midpoint of the ridge, where the implant depth is measured.
- BFP (Buccal First Point): The peripheral area (~5 mm off-center), where the cervical contour of the crown meets the surrounding soft tissue.

Due to the natural ridge curvature, the BFP is often positioned 1–2 mm more apically than the CP, particularly on the buccal side. This vertical discrepancy, known as peripheral crestal offset (PCO), must be incorporated into the final implant depth calculation to ensure a properly designed emergence profile and seamless esthetic integration.

### 4.3. Example Calculation (Including PCO Adjustment)

Consider an SPI case with

- Emergence Angle (EA) = 40°/or Flaring Angle (FA) = 50°
- Fixture Diameter = 5 mm
- Fixture–Abutment Connection (FAC) Diameter = 3 mm
- Cervical Crown Diameter = 10 mm

First, we calculate d:

D = (10 − 3)/2 = 3.5 mm

Using the emergence profile formula,

V = 3.5/tan (40) ≈ 4.17 mm

Now, adjusting for PCO (1.5 mm),

V total = v + PCO = 4.17 + 1.5 = 5.67 mm

### 4.4. Clinical Implications of V_Total

When applying this V_total clinically, clinicians must

1. Ensure that the reference point of measurement remains at the center of the edentulous crest (CP).
2. Recognize that the total vertical distance (5.67 mm) includes mucosal thickness, which varies between 2 and 3 mm.
3. Adjust implant positioning to accommodate the natural curvature of the ridge, ensuring optimal peri-implant soft tissue support.

By incorporating the PCO effect, this refined approach ensures precise implant depth determination, ultimately facilitating the achievement of a biologically stable and esthetic molar implant restoration (BEMIR).

## 5. Cross-Sectional Variations in Emergence Profiles and Their Clinical Implications from Mesiodistal Crest Slope (MDCS) Discrepancy

**Figure 2:**
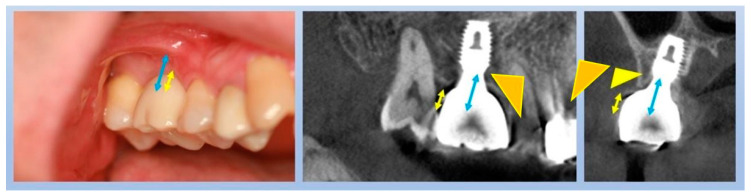
The blue arrow represents the total vertical height measured from the zenith of the edentulous mucosa at the center of the crestal ridge (CP site) to the fixture–abutment connection (FAC). The yellow arrow illustrates the vertical height difference between the zenith of the edentulous mucosa at the center of the ridge and the zenith of the edentulous mucosa at the buccal periphery, demonstrating the effect of the peripheral crestal offset (PCO). This difference accounts for the natural apical positioning of the peripheral crest relative to the central ridge. Additionally, two distinct angular measurements are illustrated by triangles—one in the buccolingual (BL) cross-section, expressed by a **thin yellow triangle**, and the other in the mesiodistal (MD) cross-section, expressed by a **thick yellow triangle**. These angular measurements represent the flaring and emergence angles from the respective cross-sectional views, which influence the emergence profile and the overall implant restoration design.

Figure 2 highlights the discrepancy between the buccolingual (BL) and mesiodistal (MD) cross-sections in emergence profiles, which differ in vertical height and emergence slope:

- BL Cross-Section: The peripheral crestal offset (PCO) causes an apical shift of the peripheral crest, increasing the total vertical height at the center point (CP). However, the vertical height from the fixture–abutment connection (FAC) to the buccal first point (BFP) is lower than that from the FAC to the CP.
- MD Cross-Section: The mesiodistal crest slope (MDCS) discrepancy results in a saddle-shaped ridge, particularly between adjacent teeth. This leads to a greater vertical height from the FAC to the mesial first point (MFP) or distal first point (DFP) compared to the FAC to CP.

Figure 2 illustrates the depth of the implant fixture in both the mesiodistal and buccolingual aspects, highlighting the differences in the zenith levels of the outer mucosal surface from each perspective. The blue arrow represents the total vertical height, measured from the zenith of the edentulous mucosa at the center of the crestal ridge (CP site) to the fixture–abutment connection (FAC). The yellow arrow indicates the vertical height difference between the zenith of the edentulous mucosa at the center of the ridge and the zenith at the buccal periphery, demonstrating the effect of the peripheral crestal offset (PCO). To achieve a biologically stable and esthetic molar implant restoration (BEMIR), the reference for implant placement depth is the most peripheral point of the restoration in each aspect—buccolingual and mesiodistal. The yellow arrow specifically illustrates the discrepancy in vertical positioning between these aspects, while the blue arrow represents the total depth of implant placement, particularly when the middle of the crest serves as the reference point for surgeons during surgery. The total depth of placement also includes mucosal thickness, which must be accounted for when the mucoperiosteal flap is reflected during implant placement surgery. This adjustment ensures a more precise estimation of the final implant position relative to the bone and overlying soft tissue, ultimately contributing to enhanced peri-implant tissue stability.

### 5.1. Key Considerations: The Influence of MDCS Discrepancy on Flaring Angle (FA) and Emergence Angle (EA)

1. MD Cross-Section Bias in Vertical Height Determination
  ο Clinicians traditionally rely on MD cross-sectional views from plain X-rays, which can lead to overestimated vertical height due to the naturally higher MD plane compared to the BL plane.
2. Impact on Flaring and Emergence Angles
  ο Since the BL cross-section requires a shorter vertical height, the flaring angle (FA_bl) is lower than the FA_md in the MD plane.
  ο Using a single emergence angle (EA) or flaring angle (FA) for both planes can cause discrepancies in soft tissue adaptation and prosthetic contouring.
3. Clinical and Prosthetic Implications
  ο Submucosal Contour Estimation: Differentiating between FA_bl and FA_md is essential to avoid errors in soft tissue adaptation.
  ο Prosthetic Design Adjustments: The buccal aspect should serve as the primary reference for esthetics, ensuring a harmonious emergence profile.

### 5.2. How Deep Is Deep Enough? Rethinking the Optimal Subcrestal Implant Depth Through Calculated Analysis

Therefore, considering that FA_bl is lower than FA_md, clinicians should reconsider the favorable EA range, which typically falls within 30–40° (corresponding to FA 60–50°). However, most clinical reports on EA originate from EA_md, as they are primarily derived from plain X-ray data. Given that EA_bl tends to be larger than EA_md in the same tooth case, as illustrated in Figure 2, it is prudent to reevaluate the clinically favorable range of EA_bl, which is likely within 60–50° (FA 30–40°).

Assuming PCO = 1.5 mm, the following estimations can be made:

- FA = 30°, v = 2.02 mm, V total = 3.52 mm
- FA = 40°, v = 2.94 mm, V total = 4.44 mm
- FA = 45°, v = 3.5 mm, V total = 5.0 mm

### 5.3. Clinical Implications

In summary, when FA_bl falls within the range of 30–45°, the corresponding V total ranges from 3.52 to 5.0 mm.

If the mucosal thickness is assumed to be 2.0 mm, the actual subcrestal implant placement depth will range between 1.52 and 3.0 mm, averaging approximately 2.0 mm subcrestal placement.

(Note: The reference point for implant placement depth is typically measured at the buccal aspect in clinical settings.)

While many clinicians may expect the proposed subcrestal placement depth to be significantly deeper than conventional practice, this analysis provides a logically derived range, demonstrating that deeper placement is not only feasible but biologically advantageous for long-term peri-implant stability in BEMIR.

While scientific data suggest that a favorable emergence angle exists—primarily derived from mesiodistal analyses, which have inherent limitations—the true clinical relevance of the emergence angle lies in its role in protecting peri-implant soft tissue. In this context, the thickness of the supracrestal tissue height (STH) becomes a critical factor for long-term implant stability. However, incorporating these parameters into clinical practice remains challenging, as it is impractical to measure precise values for every case. This is where the Three-Dimensional Soft Tissue Analysis (3DSTA) model provides a clinically beneficial approach—allowing for a biologically favorable implant restoration design that enhances peri-implant soft tissue health while optimizing esthetics.

### 5.4. Accounting for MDCS Discrepancy in Implant Placement and Radiographic Interpretation

- Surgical Considerations: MDCS discrepancy does not require adjustments during implant placement, as depth determination is primarily guided by the buccal aspect.
- Radiographic Interpretation: MDCS can cause implants to appear deeper when assessed from MFP or DFP, underscoring the importance of evaluating implant depth relative to the CP site.
- Prosthetic Adaptation: In cases with significant MDCS discrepancy, contour modifications may be necessary to ensure optimal soft tissue integration and papilla formation.

**Figure 3:**
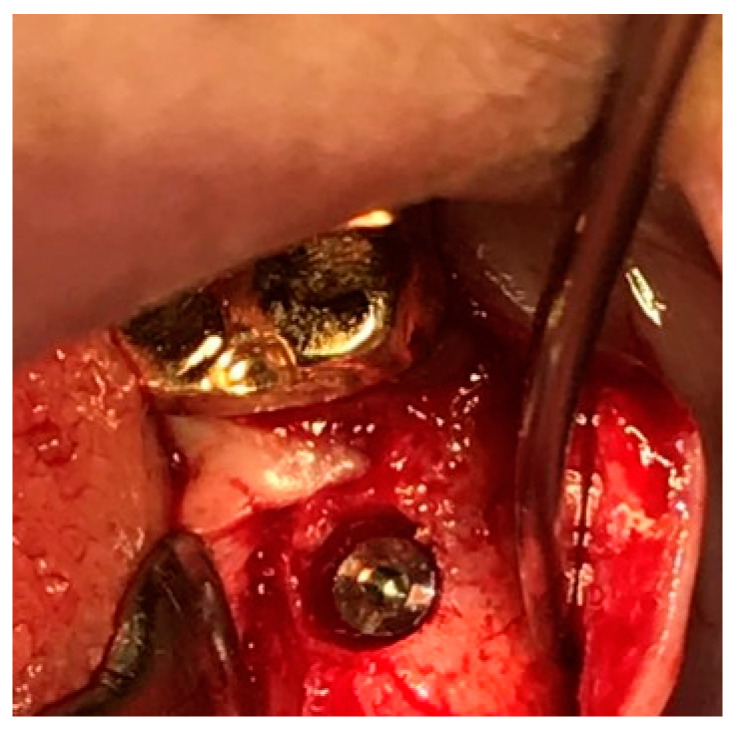
This illustration depicts the saddle-shaped edentulous ridge, where an implant is placed subcrestally while accounting for the effects of peripheral crestal offset (PCO) and mesiodistal crestal slope (MDCS). These anatomical considerations influence implant depth and emergence profile, ensuring biologic stability and esthetic integration.

By incorporating PCO, MDCS discrepancy, FA_bl, and FA_md into emergence profile planning, clinicians can achieve predictable esthetic outcomes, enhanced functional stability, and accurate postoperative assessments while mitigating errors in radiographic interpretation (Figure 3).

By recognizing the impact of MDCS on radiographic interpretation, clinicians can prevent misjudgments in assessing implant depth and ensure accurate post-surgical evaluation of implant positioning.

Additionally, the vertical distance calculated above includes the overlying mucosal thickness, typically around 2.0 mm. Since this soft tissue layer is not distinctly visible on X-ray imaging, clinicians must factor it into their surgical planning. Neglecting mucosal thickness may lead to miscalculations in actual implant depth, potentially compromising both esthetic and functional outcomes.

## 6. Clinical Scenarios for SPIs

The SPI technique is particularly advantageous in three primary scenarios:

1. Esthetic Considerations in Molar Restorations
  An SPI is frequently employed to meet esthetic demands, particularly in molar restorations, where establishing an appropriate emergence profile is essential. This technique enables seamless integration of prosthetic components with the surrounding soft tissue, enhancing both functionality and esthetics by ensuring adequate vertical soft tissue height for the transit area (transitional zone, TZ).
2. Managing Narrow Alveolar Ridges
  In cases of narrow ridges, an SPI offers an effective solution by allowing for deeper implant placement while promoting a favorable peri-implant phenotype. This ensures sufficient bone and soft tissue support, both of which are essential for long-term implant stability and health.
3. Implant Placement Adjacent to Natural Teeth
  An SPI is particularly useful in anterior or premolar regions adjacent to healthy natural teeth, especially in the upper arch. These regions often present challenges due to variations in edentulous ridge levels between buccolingual and mesiodistal interproximal spans. By placing implants subcrestally, clinicians can better accommodate these anatomic differences, preserving the functionality and esthetics of neighboring teeth.

**Figure 4:**
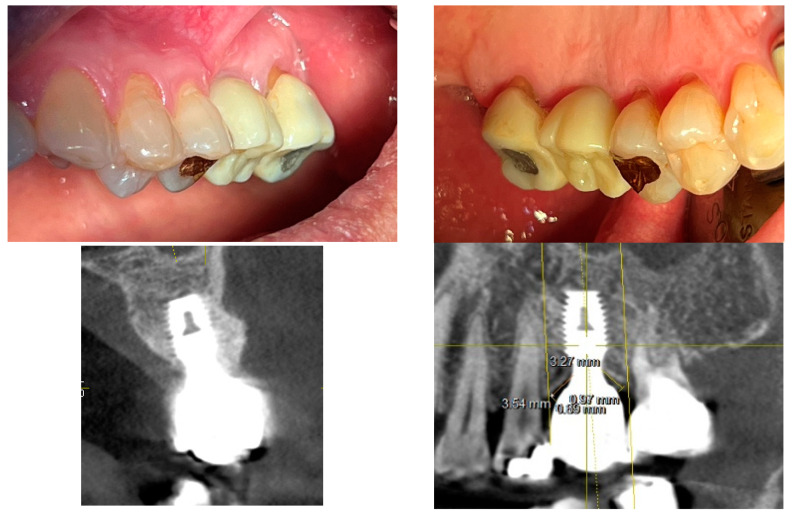
Clinical and radiographic outcomes of an SPI-restored upper left first molar. Upper Two Images (Clinical Photos): Show the restored upper left first molar with well-integrated soft tissue, demonstrating a natural emergence profile and coronal flaring of the implant restoration. These features contribute to both biological stability and esthetic success. Lower Left Image (CBCT Cross-Section, Buccolingual View): Displays the implant placement. While the PCO effect suggests that the buccal margin is positioned more apically than the mesiodistal margin—resulting in a shorter overall distance from the fixture–abutment connection (FAC) to the soft tissue margin in the buccal aspect—this does not necessarily imply that the crestal zenith at the buccal side must always be positioned apically. Unlike the palatal side, where the crest may be observed at an epicrestal level, implant placement at the buccal side can still be subcrestal, provided it meets the required vertical depth for peri-implant stability or is intended to enhance the bone phenotype. Lower Right Image (CBCT with Measurements): Highlights the transitional zone length (TZL) and soft tissue thickness (STT), showing the vertical and horizontal dimensions of the peri-implant soft tissue. Additionally, it reflects the influence of the mesiodistal crestal slope (MDCS), emphasizing how ridge morphology affects implant positioning, peri-implant soft tissue stability, and overall emergence profile.

Figure 4 showcases a successful case of SPIs, demonstrating stable and esthetic outcomes. The clinical photos highlight natural soft tissue integration, while the CBCT images provide peri-implant soft tissue analysis, including measurements of transitional zone length (TZL) and soft tissue thickness (STT). These parameters help to evaluate the relationship between structural support and biological stability achieved with SPIs.

### 6.1. Concerns About Pocket Formation in SPIs

A common concern with SPIs is the risk of pocket formation around deeply placed implants, which may compromise peri-implant health [5,6,7]. Much of this concern stems from the lack of a dedicated biologic width (or STA) model for implants. Instead, the conventional biologic width concept—originally developed for natural teeth—has been broadly applied to implants without significant modifications, often leading to misinterpretations.

### 6.2. Biologic Width (or STA) in Natural Teeth vs. Peri-Implant Tissues

The Concept of Biologic Width (or STA) in Natural Teeth

The biologic width (or STA), first described in 1961 [8], refers to the gingival soft tissue extending from the gingival sulcus to the alveolar crest. This dimension averages approximately 2.0 mm in width and is composed of two key layers:

- Junctional Epithelium (~0.97 mm wide): Functions as a protective epithelial barrier against microbial invasion.
- Connective Tissue (~1.07 mm wide): Provides mechanical support and contributes to immunologic defense mechanisms [9].

Together, these structures act as a biological seal, preventing bacterial infiltration and ensuring periodontal stability [10].

### 6.3. Structural Differences Between Peri-Implant Tissues and Natural Teeth

Although the biologic width (or STA) concept has been applied to implants, several key structural differences highlight the need for a dedicated model for peri-implant tissues [11]:

1. Absence of a Periodontal Ligament
  Natural teeth rely on the periodontal ligament (PDL) for biological and mechanical mediation. In contrast, implants achieve stability through direct osseointegration, lacking any ligamentous attachment. This structural difference increases the vulnerability of peri-implant soft tissues to mechanical stress and bacterial challenges.
2. Lack of Dentogingival Fiber Attachment
  In natural teeth, dentogingival fibers anchor the gingiva to the cementum, contributing to soft tissue stability and resistance against external forces. Implants, however, lack this intrinsic fibrous attachment, making their soft tissue seal inherently different. Peri-implant tissues rely solely on their own structural and immunological integrity to provide protection.
3. Distinct Crown–Root Morphology and Coronal Flaring
  ο Natural teeth exhibit a gradual transition from the root to the crown, allowing for smooth adaptation of the soft tissue.
  ο Implant restorations, on the other hand, display a distinct coronal flaring, where the prosthetic crown widens abruptly. This poses unique challenges for peri-implant soft tissue adaptation and stability.

Since implant restorations interface directly with peri-implant soft tissue, their morphology significantly influences soft tissue behavior. Unlike natural teeth, which have a gradual emergence profile, implants require careful emergence profile design to optimize biological stability. Consequently, multiple studies have examined the relationship between peri-implant soft tissue stability, crestal bone health, implant positioning, and restoration morphology [12,13,14,15].

### 6.4. Implications of Coronal Flaring and the Limitations of Existing Biologic Width Models

SPIs are almost exclusively performed using implants with internal platform switching (IPS) design, which offers several biological advantages, particularly in maintaining crestal bone stability. A key characteristic of SPI restorations is coronal flaring, a feature that significantly influences both soft tissue adaptation and esthetic integration [16,17,18].

However, this coronal flaring phenomenon creates a distinct peri-implant soft tissue configuration, fundamentally different from the traditional biologic width (or STA) model of natural teeth. Unlike natural teeth, where the dentogingival fibers provide intrinsic support, peri-implant soft tissues must adapt to abrupt diameter changes. This adaptation occurs primarily in the transit area—a critical horizontal and vertical zone that must be carefully designed to ensure peri-implant health and long-term stability.

Thus, the traditional biologic width (or STA) model fails to adequately explain the peri-implant soft tissue interface in three dimensions. A new model is necessary—one that accounts for

✓ Soft tissue adaptation to coronal flaring
✓ The role of the transitional zone (TZ) and subcrestal zone (SZ)
✓ Horizontal and vertical components of the peri-implant soft tissue seal

**Figure 5:**
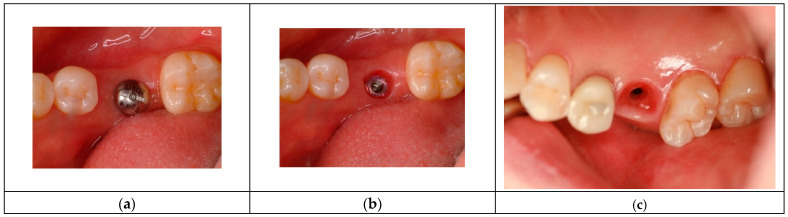
Comparison of peri-implant soft tissue architecture with and without horizontal extension. This figure illustrates three different peri-implant soft tissue configurations, highlighting the impact of implant design on soft tissue adaptation: (**a**) Left Image (Healing Abutment): Displays a healing abutment in place, where the peri-implant soft tissue adapts primarily in a vertical manner, similar to the biologic width observed around natural teeth. (**b**) Middle Image (External Hex Connection Implant): Shows an external hex connection implant embedded in the edentulous ridge without being covered by a healing cap or cover screw. In this configuration, the peri-implant soft tissue exhibits only a vertical component, directly interfacing with the implant structure in a manner akin to natural teeth. (**c**) Right Image (Internal Platform Switching Implant, SPI): Demonstrates an SPI with an internal connection and platform switching, exposed in the same manner as the middle image. Unlike the external hex implant, the peri-implant soft tissue here exhibits both vertical and horizontal components, adapting to the coronal flaring of the restoration. This horizontal extension alters the peri-implant soft tissue structure, making it more complex than the simple vertical adaptation seen in external hex implants or healing abutments. This comparison underscores the fundamental difference between traditional biologic width models based on natural teeth and the peri-implant soft tissue configuration in SPIs, necessitating a revised conceptual framework for understanding peri-implant tissue adaptation.

The images in Figure 5 illustrate the differences in peri-implant soft tissue adaptation based on implant connection type and coronal morphology.

The left-side images display cases using older external hex connection implants, where healing abutments are positioned without horizontal widening at the tissue–restoration interface. In these instances, the peri-implant soft tissue closely mimics the biologic width (or STA) observed around natral teeth, adapting vertically without significant horizontal extension. Consequently, the tissue thickness in these cases primarily reflects the dimensions of the adjacent mucosal tissue.

In contrast, the right-side images depict subcrestally placed implants (SPIs) with internal connections and platform-switching designs, which introduce a coronal flaring effect at the restoration interface. This horizontal extension at the tissue–implant junction modifies the peri-implant soft tissue structure, leading to the formation of both horizontal and vertical soft tissue components. This dimensional adaptation is a defining characteristic of peri-implant tissues influenced by coronal flaring, fundamentally distinguishing them from the soft tissue architecture seen around natural teeth and conventional external hex implants.

### 6.5. Clinical Implications of Coronal Flaring and Soft Tissue Adaptation in SPIs

**Figure 6:**
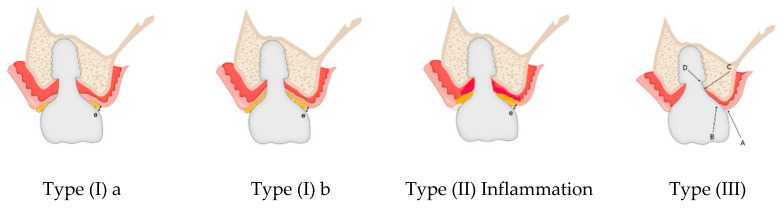
Schematic models postulated to address concerns about the development of unhealthy pockets around an SPI. What defines the transitional zone in an SPI? Unlike the biologic width (or STA) in natural teeth, the peri-implant soft tissue around an SPI is shaped by a horizontal transit zone extending from the implant–abutment connection to the outer soft tissue margin. Two plausible models describe how epithelium and connective tissue might distribute within this zone: **Type I**: Epithelium and connective tissue exist in two distinct layers throughout the entire transit zone—epithelium faces the restoration, while connective tissue contacts the crestal bone. **Type II**: Inflammation stage: As bacteria invade the sulcus, peri-implant soft tissue breakdown and inflammation begin. **Type III**: Epithelium is present only at the entrance of the transit zone, while the remainder consists of a single-layer connective tissue barrier. Determining factors for either model likely depends on restoration design, crestal bone topography, and peri-implant soft tissue thickness and length. A–B indicates the area occupied by sulcular epithelium; B–C indicates the transitional zone (TZ), consisting of junctional epithelium and connective tissue; C–D represents the subcrestal zone (SZ); e represents a void space.

The horizontal extension created by the platform-switching design of SPIs and coronal flaring plays a crucial role in reinforcing peri-implant soft tissue stability. To construct a model, this inevitably generated and observed zone serves as the starting point for investigating and explaining these circumstances. Thus, it is essential to consider the differences between natural teeth and implants, as well as the differences between older implant designs, which lacked significant horizontal extensions, and modern SPIs. When peri-implant soft tissue interfaces with the submucosal part of the abutment and restoration over a broader area—without the attachment apparatus seen in natural teeth—concerns naturally arise regarding the potential for a perilous pocket, similar to those observed in periodontally compromised teeth. To explain this horizontal extension area, three possible types can be proposed, as illustrated in Figure 6:

Type I: The connective tissue is confined to the deep area around the fixture–abutment connection (FAC), while the majority of the horizontal extension is covered by epithelial cells, primarily the sulcular epithelium.

Type I-a: The epithelial layer is in direct contact with the submucosal part of the abutment and restoration, except at the entrance area, where a sulcus may exist as a void space.

Type I-b: The epithelial layer remains in contact with the submucosal part of the abutment and restoration only in the inner region near the FAC, while the majority of the area remains void, with no epithelial attachment to the abutment surface.

Type II (Inflammation Stage): This represents a stage where the tissue attempts to fill the void created in Type I, leading to an inflammatory response as the body fights against bacterial invasion.

Type III (Optimal State): In this scenario, the majority of the horizontal area is occupied by connective tissue (with some junctional epithelium at the peripheral area) in close contact with the submucosal part of the abutment and restoration. Sulcular epithelial cells are limited to the entrance area, ensuring a stable peri-implant soft tissue interface. Compared to Type I, this configuration is far less prone to inflammation, even with routine patient oral hygiene.

From a histological perspective, the cellular layers, their dimensions, and their structural organization each have characteristic sizes. The determinant for the formation of Type I or Type III depends on the available space between the underlying crestal bone and the submucosal part of the abutment and restoration—referred to as the crest to restoration distance (CRD). When the CRD exceeds a critical range, Type I is inevitable due to the structural necessity of the sulcular epithelium, which requires a minimum volume to maintain its integrity. When CRD remains within a critical thickness, Type III is expected based on the known histological properties of connective tissue and epithelium. In Type III, the CRD is fully occupied by soft tissue, with no voids. Therefore, in this scenario, CRD can be directly measured as soft tissue thickness (STT).

The connective tissue and junctional epithelium in this region are considered stable and self-sustaining, without requiring coverage by the sulcular epithelium for protection from the external environment. This transitional zone, located in the supracrestal area, exhibits histological characteristics that are distinct from conventional biologic width (or STA). Similarly, connective tissue with self-sustaining properties in the subcrestal area is designated as the subcrestal zone (SZ). Since junctional epithelium shares similarities with connective tissue in its histological structure and can exist as a single-layer component, the transitional zone (TZ) may be composed of both connective tissue and junctional epithelium.

### 6.6. Why This Analysis Is Critical for 3DSTA

Understanding the histologic variations within the peri-implant transit zone is crucial for establishing a clinically relevant framework for evaluating SPI restorations. By considering these hypothetical models, 3DSTA can provide a structured approach to analyzing peri-implant soft tissue in three dimensions, bridging the gap between theoretical morphology and real-world clinical adaptation.

**Figure 7:**
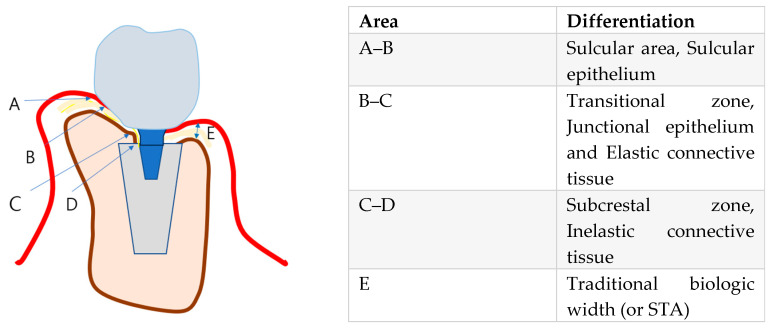
Schematic drawing to differentiate the peri-implant soft tissue into 3 zones: (1) sulcular area, (2) transitional zone, and (3) subcrestal zone.

Figure 7 is a schematic representation of peri-Implant soft tissue zones. This schematic drawing illustrates the differentiation of peri-implant soft tissue into three distinct zones, each playing a crucial role in the biological stability and function of SPI:

### 6.7. Peri-Implant Soft Tissue Zones

- **Sulcular Area**—The most coronal portion, adjacent to the restoration, lined with the sulcular epithelium. This zone is functionally similar to the gingival sulcus around natural teeth, permitting limited probing without compromising the biological seal. In cases where a healthy transitional zone (TZ) is established, the probe tip naturally stops within the sulcus, indicating the integrity of the peri-implant soft tissue barrier.
- **Transitional Zone (TZ)**—The horizontal extension of peri-implant soft tissue that connects the sulcular area to the deeper subcrestal zone. This region is hypothesized to contain both junctional epithelium and connective tissue, forming a critical biologic barrier against external irritants. Structurally, the TZ is essential for stabilizing peri-implant soft tissue and preventing apical migration of the epithelium, thereby maintaining peri-implant health.
- **Subcrestal Zone (SZ)**—The portion of peri-implant soft tissue located below the crestal bone level. This zone is predominantly composed of connective tissue and remains relatively thin due to its direct proximity to the subcrestal bone.

Histological Perspective on Junctional Epithelium and Connective Tissue: Addressing the Differences Between Implants and Natural Teeth for Clinical Advantage.

While biologic width (or STA) in natural teeth is well established, the horizontal transit layer in SPIs remains less clearly defined, raising questions about its structural composition and biological function. Since direct histologic examination of peri-implant tissue is impractical in a clinical setting, logical postulation based on known tissue behavior is required.

### 6.8. Connective Tissue and Its Role in Peri-Implant Health

Connective tissue within the peri-implant soft tissue plays a critical role in maintaining biological stability. Its thickness and proximity to the underlying crestal bone, as measured through crest to restoration gap (CRG)—which transitions into soft tissue thickness (STT) when optimized—are key to its structural and functional integrity. Maintaining stable CRG/STT dimensions is essential for long-term peri-implant health, preventing bone loss, and supporting soft tissue adaptation.

**Key Functional Roles of Peri-Implant Connective Tissue:**

- Supporting the Junctional Epithelium—Provides a stable foundation and vascular supply, ensuring biological sealing.
- Immune Functionality—Acts as a conduit for immune cells and signaling molecules, facilitating immune responses.
- Mechanical Sealing—Resists external forces through resilience and biochemical properties, including protein–carbohydrate macromolecules.
- Tissue Repair—Serves as a reservoir of stem cells and fibroblasts, promoting healing and soft tissue regeneration.
- Bone Protection—Functions similarly to the periosteum, safeguarding the underlying bone and promoting homeostasis.

### 6.9. Junctional Epithelium and Its Role in Peri-Implant Soft Tissue Stability

The junctional epithelium is the first line of defense against microbial colonization around implants. While its sealing capacity is reported to be less robust than that of natural teeth, hemidesmosomal attachments have been well documented, forming a functional barrier by adhering both to the implant surface and the underlying connective tissue [19,20,21,22,23,24].

**Key Features of Peri-Implant Junctional Epithelium:**

- Limited Thickness—Typically 0.5–1 mm, this layer is restricted to the coronal portion of the peri-implant soft tissue, while connective tissue comprises the majority of the interface.
- High Turnover Rate—The rapid renewal capacity of the junctional epithelium aids in maintaining its protective barrier.
- Structural Characteristics—Consists of 15–30 cell layers with larger intercellular spaces and fewer desmosomes compared to oral epithelium. This feature enhances immune cell mobility, improving immunological function.
- Hemidesmosomal Attachments—These structures provide adhesion to both the implant surface and the underlying connective tissue, forming a biologic seal that prevents microbial invasion. While weaker than the attachment seen in natural teeth, it remains essential for peri-implant integrity.
- Immunological Function—Facilitates endocytosis and decomposition of exogenous factors, helping to regulate the local immune response.

### 6.10. Need for 3-Dimensional Analysis

By understanding these fundamental histological differences, clinicians can optimize peri-implant tissue behavior through prosthetic design, surgical techniques, and strategic soft tissue management to enhance peri-implant health and longevity.

**Figure 8:**
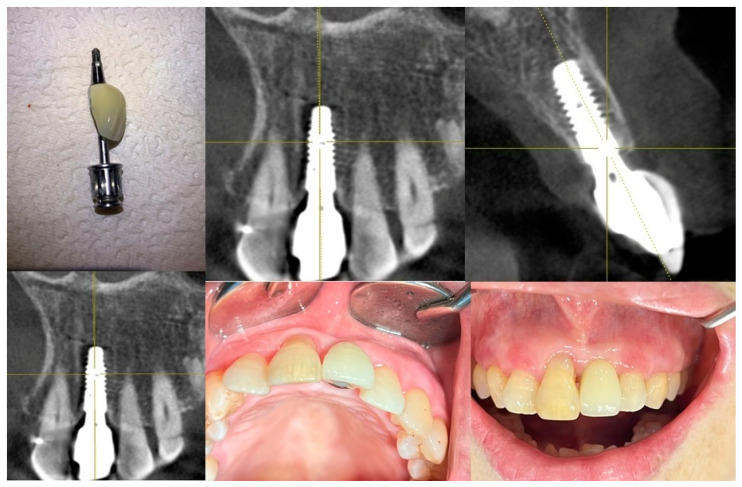
Although the plain X-ray image suggests that the implant was placed subcrestally, the CBCT image reveals that the fixture was actually placed epicrestally in the buccolingual aspect. This discrepancy highlights the limitations of two-dimensional imaging, emphasizing the need for three-dimensional evaluation to accurately assess implant positioning relative to the crestal bone in both the buccolingual and mesiodistal dimensions.

Figure 8 illustrates a common clinical observation: while the plain X-ray suggests a subcrestal implant placement, the CBCT image reveals an epicrestal position in the buccolingual aspect. This discrepancy highlights the limitations of two-dimensional imaging and reinforces the necessity of three-dimensional evaluation for precise implant positioning.

### 6.11. Variations in Peri-Implant Soft Tissue Adaptation Based on Implant Placement Depth

1. **Epi- or Equicrestal Placement without Submucosal Flaring**

The implant restoration maintains a gap (e) between the cervical contour and the underlying soft tissue, preventing tissue compression but resulting in a non-anatomic emergence profile. Here, (a) represents the thickness of the epithelium and connective tissue, analogous to the biologic width (or STA) in natural teeth.

2. **Epi- or Equicrestal Placement with Submucosal Flaring**

The buccal cervical contour of the restoration maintains direct contact (f) with the underlying mucosa, creating a more natural emergence profile. However, adequate buccal bone support is required to prevent soft tissue recession or marginal bone loss due to excessive pressure on the mucosa.

3. **Subcrestal Implant Placement (SPI) with Transitional Zone (TZ) Formation**

The implant is positioned subcrestally, leading to complex peri-implant soft tissue adaptation with both vertical and horizontal components. The transitional zone (TZ) serves as a biologic interface, contributing to peri-implant stability and soft tissue integration. This schematic also introduces key variables that will be discussed in detail later:

- a, e, f—Same as in (1) and (2).
- b, c—Representing soft tissue thickness (STT).
- d—Representing crest to restoration length (CRL).
- g—Representing free-standing peripheral soft tissue height.

**Figure 9:**
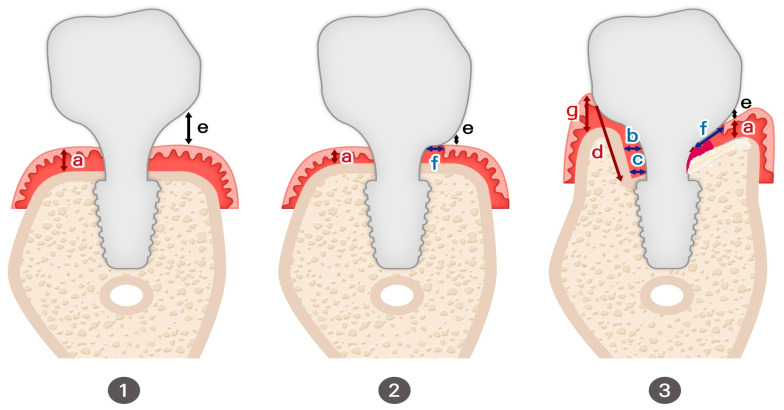
Varia Variations in peri-implant soft tissue adaptation based on implant placement depth. In this schematic illustration, a represents the conventional biologic width (supracrestal tissue attachment; STA). b and c represent the thickness of self-sustained soft tissue (SSST) at each corresponding area. d represents the total length of SSST, which is composed of the transitional zone length (TZL) and subcrestal zone length (SZL). f represents TZL, while g represents the total supracrestal tissue height at the peripheral area. e represents the void space.

Figure 9 presents three distinct peri-implant soft tissue configurations, demonstrating how different implant placement depths and emergence profile designs influence soft tissue stability, biologic integration, and esthetic outcome.

1. **Epi- or Equicrestal Placement without Submucosal Flaring**

In this scenario, the cervical contour of the restoration does not extend beneath the mucosa, avoiding soft tissue compression by keeping the flaring region entirely supramucosal.

- This results in a void space (e) between the restoration and mucosa, reducing peri-implant soft tissue adaptation but preventing excessive compression.
- The soft tissue thickness (a) corresponds to the traditional biologic width (or STA) model in natural teeth but lacks the connective tissue attachment characteristic of natural dentition.
- Due to the absence of horizontal flaring, the emergence profile appears unnatural, with the crown contour remaining narrow until it exits the mucosa.
- This design is commonly seen in healing abutment placement and external hex connection implants, where peri-implant soft tissue adaptation occurs only in a vertical dimension.

2. **Epi- or Equicrestal Placement with Submucosal Flaring**

This variation incorporates a submucosal flaring design by allowing the buccal cervical contour of the crown to maintain direct contact with the underlying mucosa, mimicking the emergence profile of natural teeth.

- The soft tissue contact distance (f) represents the region where the restoration interfaces with the peri-implant mucosa.
- This configuration provides a more natural emergence profile, but results in a more abrupt emergence compared to SPI.
- However, buccal bone thickness is a critical determinant of soft tissue stability—insufficient buccal bone support may increase the risk of tissue recession or marginal bone loss due to excessive pressure on the mucosa.
- Clinicians must carefully balance flaring dimensions and soft tissue adaptation to achieve optimal esthetic and biological outcomes, as a reduced emergence angle has been associated with unfavorable peri-implant soft tissue responses.

3. **Subcrestal Implant Placement (SPI) with Transitional Zone Formation**

In SPI cases, the subcrestal positioning of the implant leads to a more complex peri-implant soft tissue adaptation, where both vertical and horizontal components influence the biological interface.

- Unlike epi- or equicrestal placements, the horizontal extension of the soft tissue beneath the mucosa generates a distinct transitional zone (TZ).
- This horizontal adaptation cannot be adequately explained using the traditional biologic width (or STA) model, as it introduces new tissue dynamics that influence emergence profile stability.
- This peri-implant soft tissue adaptation is shaped by
  ο The relationship between the restoration and underlying bone
  ο The width and height of peri-implant soft tissue
  ο The degree of subcrestal positioning
  ο The interplay between connective tissue and junctional epithelium in the TZ

## 7. Implications for 3DSTA

These three scenarios highlight the necessity for a three-dimensional soft tissue analysis (3DSTA), as traditional two-dimensional models fail to account for the horizontal components of peri-implant soft tissue adaptation.

- In equi- or epicrestal placements, peri-implant soft tissue remains vertically oriented, allowing a simplified emergence profile evaluation.
- In SPI cases, the transitional zone (TZ) adds a horizontal dimension, requiring a more nuanced analytical approach to understand its biologic function.
- The 3DSTA model provides a systematic method to quantify peri-implant soft tissue behavior, ensuring predictable soft tissue adaptation and long-term implant stability.

By incorporating 3DSTA principles, clinicians can strategically design peri-implant soft tissue contours, ensuring optimal esthetic integration, biologic sealing, and functional longevity in implant restorations.

### 7.1. New 3-Dimensial Model (3DSTA: 3-Dimensional Soft Tissue Analysis)

**Figure 10:**
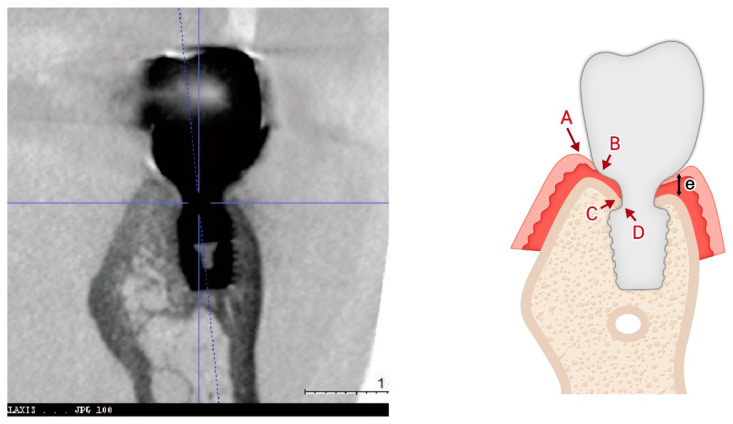
Three-dimensional schematic model for 3D soft tissue analysis (3DSTA). This model, based on a real CBCT image (**left**), illustrates the key components of peri-implant soft tissue in a subcrestally placed implant (SPI). The schematic (**right**) highlights three zones: the sulcus zone (A–B), transitional zone (TZ, B–C), and subcrestal zone (SZ, C–D). The traditional biologic width (or STA) (e), observed in equicrestal implants, is shown for comparison. This model provides a framework for measuring peri-implant soft tissue dimensions using CBCT.

Figure 10 introduces a three-dimensional soft tissue analysis model (3DSTA) designed to quantify peri-implant soft tissue components using CBCT imaging. The left image represents a real CBCT scan, providing the foundation for the schematic. The right schematic model is derived from the CBCT image outlining the soft tissue structure around subcrestally placed implants (SPIs).

### 7.2. New Concepts with Core Hypotheses

A summary of the peri-implant soft tissue parameters, distinguishing measurable components from inferred components and indicating their visibility in CBCT imaging, is provided in Table 2.

### 7.3. Distinguishing Crest to Restoration Distance (CRD), Soft Tissue Thickness (STT), and Self-Sustained Soft Tissue (SSST)

The crest to restoration distance (CRD) represents the vertical distance measured from the crestal bone to the restoration interface, encompassing void spaces, epithelial layers, and connective tissue. However, the CRD transitions into soft tissue thickness (STT) only when it lies within an optimal thickness range—a hypothetical proposal grounded in clinical observations and outcomes. While the exact “optimal thickness” awaits validation through large-scale statistical investigations and further research, it is not an unvalidated concept. Rather, it carries its own identity as a valid notion derived from observed clinical outcomes.

The distinction between CRD and STT lies in their functional and biological significance. STT refers specifically to the biologically functional thickness of self-sustained soft tissue (SSST). When measured using radiographic tools such as CBCT, STT can align with CRD only if the CRD falls within the optimal range, where void spaces and epithelial layers are minimal or absent. Conversely, a CRD exceeding this threshold indicates void formation and epithelial downgrowth, which predisposes the peri-implant environment to pocket formation, bacterial infiltration, and inflammation.

Maintaining a CRD within its optimal range is crucial for fostering the formation of SSST—a biologic structure composed of the transitional zone (TZ) and subcrestal zone (SZ). SSST serves as a stable connective tissue interface that resists epithelial migration, creates a protective seal around the implant, and supports long-term peri-implant health.

**Key distinctions:**

1. **CRD** is a measurable vertical parameter influenced by implant placement depth and prosthodontic design. It reflects a composite dimension that may include voids and epithelial layers, depending on its alignment with the optimal thickness range.
2. **STT**, on the other hand, conveys the biologically functional tissue thickness of SSST. It is valid only when void spaces are absent, and the connective tissue is structurally optimized to support peri-implant health.

Although both CRD and STT can be measured radiographically, their implications differ. CRD is a descriptive vertical measurement, while STT represents a biologically functional dimension. The concept of optimal thickness, though awaiting broader validation, is firmly rooted in clinical experience and provides a meaningful framework for understanding the biological behavior of peri-implant tissues.

### 7.4. SSST in SPIs

Self-sustained soft tissue (SSST) in subcrestally placed implants (SPIs) is a biologic structure that can be conceptualized as a three-dimensional model integrating vertical and horizontal components:

- **Vertical component:** Represented by soft tissue thickness (STT), which corresponds to the optimized crest to restoration distance (CRD). This ensures sufficient connective tissue thickness to protect the peri-implant interface and maintain peri-implant health.
- **Horizontal component:** Defined by the transitional zone length (TZL), which establishes a robust biologic seal around the implant restoration.
  - In subcrestally placed implants (SPIs), the horizontal dimension of SSST is exclusively defined by the transitional zone length (TZL), which contributes to the biologic sealing function.
  - Supracrestal tissue height (STH) is traditionally defined as the vertical distance from the crest tip to the zenith of the free-standing peripheral soft tissue coronally. However, in the unique environment of SPIs, a new term—total soft tissue distance (TSTD)—is proposed to better describe this composite concept.
  - TSTD in SPIs encompasses both the original vertical distance (STH) from the crest tip to the zenith of the free-standing peripheral soft tissue and the TZL, which is essential for the biologic functionality of SSST.
  - In SPIs, TZL serves as the primary horizontal connective tissue component, while the free-standing peripheral soft tissue height reflects the coronal soft tissue extending beyond the constricted and contained environment surrounded by the underlying crestal bone, adjacent implant restorations, or neighboring teeth.
  - TZL is difficult to measure clinically because the boundary between the sulcular epithelium and the junctional epithelium cannot be distinguished in a clinical setting. As a result, TZL is considered a histological component rather than a direct clinical parameter. However, if STT at the TZ is statistically validated, it may become possible to theoretically infer and measure TZL in clinical practice.
  - Clinically, TZL can be inferred through probing, as the junctional epithelium layer within the TZ is expected to resist penetration when an optimal probing force is applied.

**Subcomponents of SSST:**

1. **Subcrestal Zone (SZ):**

The subcrestal zone (SZ) is formed at the interface between the implant and the neighboring subcrestal bone when the implant is placed subcrestally. Despite its clinical importance, the existence of this zone has historically been overlooked. Early implant research and clinical practice intentionally neglected SZ because it was assumed that this area must achieve osseointegration, and any soft tissue engagement was considered undesirable or contrary to the ideal of osseointegration. Fibro-osseointegration was seen as contradictory to the foundational principle of implant success.

However, long-term clinical observations have demonstrated that this zone exhibits remarkable stability, even in the absence of strict osseointegration. This phenomenon is particularly noticeable in bone-level implants placed deeply subcrestally, where restorations are designed to maintain an intimate relationship with the neighboring subcrestal bone without cutting or removing it. This stability is especially evident when matching abutment techniques are employed. Based on Won’s study of 20 cases performed with SPIs incorporating internal platform switching (IPS), the estimated thickness of the SZ is approximately 0.3 mm [25].

2. **Transitional Zone (TZ):**

The transitional zone (TZ) represents the coronal elastic connective tissue zone that dynamically adapts to the implant restoration, playing a vital role in maintaining a tight biologic seal. Unlike the subcrestal zone (SZ), which is primarily a biologic response, the formation and characteristics of the TZ are predominantly influenced by clinical design and intentional prosthodontic planning.

The TZ is shaped by

- The overall size and contour of the restoration
- The width and anatomy of the surrounding crestal bone

The thickness of the TZ, represented as soft tissue thickness (STT) at the TZ, theoretically ranges from 0.3 mm to 1.5 mm in width, depending on the restoration design and its interaction with the crestal bone. However, further studies are necessary to validate these measurements and investigate their clinical implications. Acting as an elastic connective tissue zone, the TZ ensures a secure biologic seal around the restoration, helping to protect underlying peri-implant structures.

Further studies are needed to clinically investigate the STT of the TZ through statistical analysis. The self-stained soft tissue (SSST) begins where the junctional epithelium starts. If the crest to restoration gap (CRG) is excessive, the sulcular epithelium is present along with a void (sulcus facing sulcular epithelium). However, once the sulcular epithelium is no longer required, the junctional epithelium must begin.

**The Junctional Epithelium and Connective Tissue as a Functional Unit:**

Due to histologic similarities between the junctional epithelium and connective tissue, a single layer of junctional epithelium may extend over the connective tissue of the TZ. Given this structural and functional overlap, the junctional epithelium and the connective tissue of the TZ can be considered a single functional unit—integrating both biologic function and structural harmony in subcrestally placed implants.

**Figure 11:**
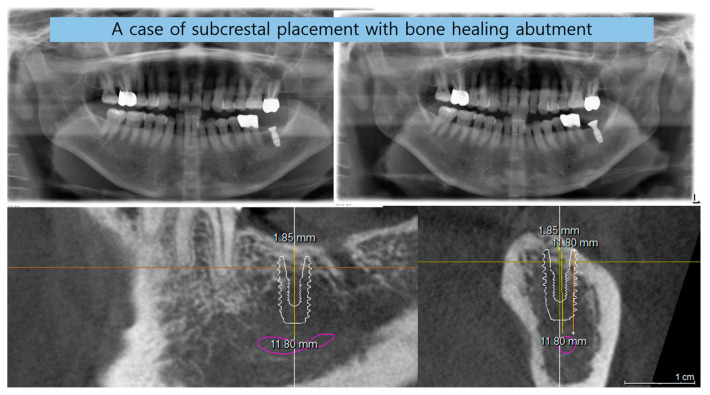

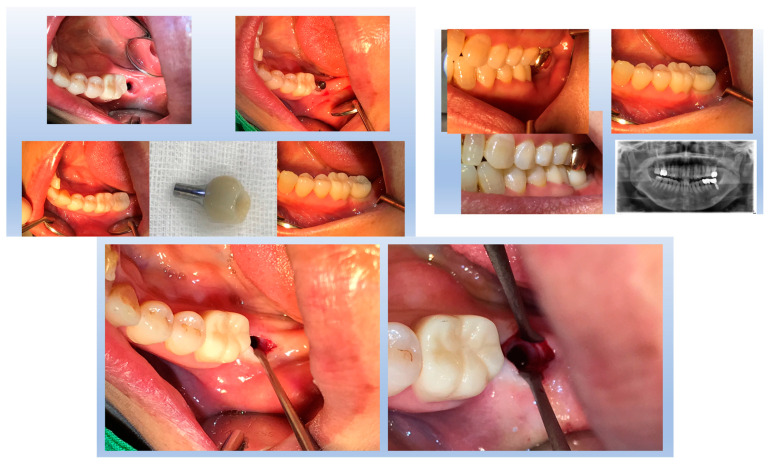
Procedural steps for matching ready-made abutments with subcrestally placed healing abutments. The upper images include X-rays and clinical photographs, illustrating the process of matching ready-made abutments with subcrestally placed healing abutments. The lower images highlight the transitional zone (TZ) and subcrestal zone (SZ), showing their structural and functional differences. The TZ appears pink and elastic, while the SZ is paler and less elastic. Notably, the SZ forms after using a matching healing abutment due to its proximity to the adjacent subcrestal bone. Despite its thin structure, the SZ remains functional and stable, possibly due to its protected position beneath the TZ.

Figure 11 illustrates the step-by-step process of integrating pre-fabricated abutments with subcrestally placed healing abutments and the corresponding biological response of peri-implant soft tissue.

In the upper images, both X-ray and clinical photographs capture the procedural workflow involved in this process. These images demonstrate how ready-made abutments are carefully matched with subcrestally placed implants. The application of healing abutments plays a crucial role in shaping peri-implant soft tissue, ensuring a well-adapted emergence profile that facilitates long-term soft tissue stability.

The lower images provide a closer look at the peri-implant soft tissue structure following this process. Two distinct zones can be observed: the transitional zone (TZ) and the subcrestal zone (SZ). The TZ appears pink and elastic, indicating its dynamic adaptation to the implant’s emergence profile. In contrast, the SZ is paler and less elastic, positioned closer to the crestal bone, where it serves a more structural and stabilizing role. Unlike the TZ, the SZ is not naturally present, but is formed as a result of healing abutment placement, developing due to its proximity to adjacent subcrestal bone.

Despite its thinness, the SZ remains functionally stable, likely benefiting from its deep subcrestal location, where it is protected from excessive mechanical stress. The overlying TZ may further contribute to this protection, reinforcing the structural and biological stability of the peri-implant soft tissue.

This figure underscores the importance of healing abutments in peri-implant tissue adaptation, revealing how distinct soft tissue zones develop around implants and contribute to long-term stability and biological integration.

### 7.5. Confirmation of Self-Sustained Soft Tissue (SSST) Existence

The distinct separation of the transitional zone (TZ) and subcrestal zone (SZ) in Figure 11 provides strong visual and clinical evidence supporting the concept of self-stained soft tissue (SSST). The presence of the SZ beneath the peri-implant mucosa, maintaining its stability without epithelial coverage, challenges traditional assumptions that peri-implant soft tissue must always be epithelialized for long-term functionality.

This observation aligns with clinical experiences where SZ formation is consistently observed in subcrestally placed implants (SPIs), particularly in cases where healing abutments are carefully matched. The functional integrity of the SZ—despite its thinness and subcrestal location—demonstrates that SSST can exist as a stable biological structure without requiring epithelial coverage.

These findings reinforce the hypothesis that peri-implant soft tissue does not necessarily need to be completely epithelialized to ensure biological integration and mechanical resilience. Instead, SSST, composed of the SZ and TZ, can sustain itself through connective tissue attachment and vascular support, providing an alternative biological model for peri-implant soft tissue adaptation.

This insight has profound implications for implant design, soft tissue management, and the understanding of peri-implant biologic (or STA) width in subcrestally placed implants. By acknowledging the functional presence of SSST, clinicians can optimize implant restoration strategies to harness the biological advantages of deeper soft tissue zones, ultimately enhancing peri-implant health and long-term stability.

## 8. The Peri-Implant Phenotype and the Role of SPIs

In contemporary implantology, the peri-implant phenotype is recognized as a critical factor for achieving long-term stability. Traditionally, three key components have been emphasized [26]:

1. Alveolar Bone Thickness: A minimum of 2 mm thickness around implants (buccal, lingual, and interproximal) is necessary to prevent bone loss and provide mechanical support, particularly for forces concentrated near the crestal area.
2. Width of Attached Gingiva: A thick gingival phenotype improves resistance to inflammation and mechanical stress.
3. Biologic Width: This provides a protective barrier through soft and hard tissues, ensuring long-term health.

Subcrestally Placed Implants (SPIs) supports and enhances these components:

- Alveolar Bone Thickness: By placing implants subcrestally, the fixtures are surrounded by thicker crestal bone, improving structural support and bone stability.
- Soft Tissue Reinforcement: Subcrestal placement enlarges the connective tissue area, which protects the underlying bone and creates a stable biologic barrier.
- Functional Biologic Width (or STA): SPI leverages the horizontal dimension of platform-switching designs, stabilizing soft tissue and minimizing crestal bone remodeling.

Additionally, an SPI offers esthetic benefits by creating a more natural emergence profile. The enlarged circular area of soft tissue, visible occlusally after healing cap removal, strengthens both biological and visual outcomes [27].

Traditionally, clinicians have relied on bone grafting or connective tissue grafting to improve these phenotype components in cases of epicrestal placement. An SPI provides an effective alternative by ensuring that implants are surrounded by sufficient bone and soft tissue, enhancing structural integrity and biologic function without the need for additional invasive procedures. This positions the SPI as an important technique for achieving stable and esthetic implant outcomes while addressing the essential elements of the peri-implant phenotype.

### 8.1. Clinical and Practical Implications

Recognizing the biological and structural advantages of SPIs helps clinicians optimize implant placement and restoration design. This concept is particularly relevant in the following areas:

1. Creating a Natural-Looking Emergence Profile—An SPI contributes to a seamless transition between implant restoration and surrounding soft tissue.
2. Distinguishing Self-Sustained Soft Tissue (SSST) from Pathologic Pockets—A well-adapted peri-implant soft tissue zone helps differentiate healthy peri-implant adaptation from unfavorable soft tissue pocket formation.
3. Achieving a Favorable Peri-Implant Phenotype—An SPI promotes thicker peri-implant soft tissue, reducing inflammation risk and enhancing soft tissue stability.
4. Establishing a Functional Biologic Seal—An SPI contributes to peri-implant immune defense by enhancing soft tissue sealing and preventing microbial invasion.
5. Laying the Foundation for Digital Implant Restoration Design—Understanding peri-implant tissue behavior facilitates precise restoration planning using CAD/CAM technology.
6. Determining Optimal Implant Placement Depth—An SPI allows customized depth selection, optimizing soft tissue adaptation and biomechanical performance.

### 8.2. Clinical Evidence: Biologic Width Adaptation in SPIs

**Figure 12:**
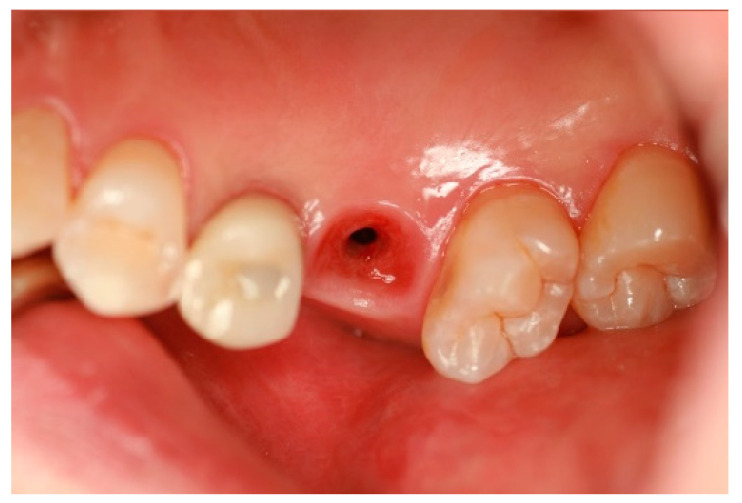
Clinical images of peri-implant soft tissue showing biologic width adaptation.

Figure 12 presents clinical images that highlight peri-implant soft tissue adaptation in SPI cases. These images were taken after the prosthetic component was removed or before placement, providing an unobstructed view of the peri-implant biologic width (or STA).

Compared to epi- or equicrestal placements, SPIs generate a larger transmucosal soft tissue area, which contributes to

- Improved peri-implant soft tissue stability
- Enhanced biologic sealing
- Greater resistance against microbial penetration

The image distinctly identifies three structural soft tissue layers:

1. Subcrestal Zone (SZ)—The deepest layer, adjacent to the crestal bone, serving as a stabilizing foundation for peri-implant soft tissue.
2. Transitional Zone (TZ)—A dynamic interface between the implant restoration and soft tissue, playing a key role in biologic width (or STA) adaptation.
3. Sulcular Area—The most coronal portion, which exhibits a pale pink color, closely resembling adjacent oral epithelium, ensuring continuity between natural and peri-implant soft tissues.

This figure underscores the distinctive biologic width (or STA) adaptation in an SPI, differentiating it from equicrestal or epicrestal placements. The formation of the subcrestal and transitional zones in an SPI provides structural and functional advantages, contributing to implant longevity and long-term soft tissue health.

These clinical images illustrate the peri-implant soft tissue structure after removal or before placement of the prosthetic component in subcrestally placed implants (SPIs). Compared to epi- or equicrestal placements, SPIs exhibit a larger transmucosal soft tissue area, contributing to enhanced soft tissue adaptation. In the right image, three distinct layers are visible: the subcrestal zone (SZ), the transitional zone (TZ), and the sulcular area, with the pale pink color of the sulcular region resembling that of the adjacent oral epithelium.

## 9. Biological and Esthetic Advantages of SPIs: Addressing Concerns About Peri-Implant Soft Tissue Stability

Subcrestally placed implants (SPIs) with internal platform switching (IPS) are widely recognized for their role in enhancing bone stability and soft tissue reinforcement [4]. While early crestal bone loss is often attributed to biologic width (or STA) accommodation, an SPI creates a favorable peri-implant environment by minimizing this adaptation and reducing microbial susceptibility. The increased surface area of the transitional zone (TZ) in an SPI facilitates a more robust soft tissue interface, lowering the risk of peri-implant diseases such as mucositis and peri-implantitis.

Palacios et al. reported that both crestal and subcrestal implant placements achieved high survival rates, with comparable bone loss. However, subcrestal placement was preferred due to its potential to reduce the likelihood of future implant exposure, which may otherwise contribute to peri-implant complications [28,29].

### 9.1. The Role of Soft Tissue Volume in Peri-Implant Stability

Several studies have demonstrated that insufficient peri-implant soft tissue volume—resulting either from shallow implant placement or a suboptimal abutment–soft tissue relationship—is associated with crestal bone loss [14,27,30,31,32,33]. This is primarily attributed to the soft tissue’s reduced ability to serve as a protective biological barrier against microbial invasion and mechanical stress.

A key parameter in SPI design is transitional zone length (TZL). According to Won’s study, the average TZL measures 3.8 mm, with an average thickness of 0.6 mm, suggesting that the majority of the TZ is composed of connective tissue. As the TZ extends peripherally, its thickness increases beyond 1.0–1.5 mm, at which point it may become covered by the junctional epithelium [25]. This aligns with Ikiru Atsuta et al.’s findings, which indicate that the junctional epithelium typically covers only the entrance area of the submucosal zone, measuring approximately 0.5–1 mm [20]. These findings reinforce the notion that connective tissue, rather than epithelium, dominates the TZ, playing a crucial role in peri-implant stability.

### 9.2. The Impact of an Enlarged Transitional Zone on Peri-Implant Health

One of the biological advantages of an SPI is its ability to increase the TZ’s surface area, providing a broader interface for blood supply from the alveolar bone. This vascularization is critical for

- Reducing peri-implant inflammation
- Enhancing resistance to bone loss
- Compensating for the lack of connective tissue attachment, which natural teeth possess via the periodontal ligament

Given that dental implants inherently lack periodontal ligament fibers, the expanded TZ in SPI cases serves as a functional adaptation, compensating for the biologic width (or STA) limitations of traditional implant designs.

### 9.3. Addressing Concerns About Long Peri-Implant Soft Tissue in SPI

While an SPI enhances both esthetics and biological stability, concerns remain regarding SSST—the long peri-implant soft tissue interface between the implant restoration and the underlying crestal bone.

A healthy SSST must prevent epithelial downgrowth and function similarly to the natural biologic width. To validate this mechanism, two key investigations are required:

1. Dimensional stability of SSST: Ensuring that peri-implant soft tissue thickness remains stable over time.
2. Sealing ability of the junctional epithelium: Verifying that the epithelium effectively prevents microbial penetration.

1. **Assessing the Stability of Peri-Implant Soft Tissue Dimensions**

The first step in addressing concerns about SSST stability is to monitor the dimensional consistency of its components. A key parameter of interest is the crest to restoration gap (CRG), which transitions into soft tissue thickness (STT) when optimized.

The findings from Won’s study indicate

- Average central soft tissue thickness (cSTT): 0.3 mm
- Average peripheral soft tissue thickness (pSTT): 0.6 mm
- Stable measurements across the observation period, indicating consistent peri-implant soft tissue dimensions

These results suggest that SSST maintains structural integrity, reinforcing its role in peri-implant health [33].

2. **Evaluating Peri-Implant Soft Tissue Integrity Through Probing**

Probing the peri-implant sulcus is a crucial technique for assessing the structural and functional stability of peri-implant soft tissues, particularly to evaluate the sealing capacity of the junctional epithelium. However, the unique anatomical features of the peri-implant sulcus, such as its curved anatomy, complicate probing. Unlike natural teeth, there are no standardized normal probing depths for subcrestally placed implants, making consistent evaluation challenging.

Traditional metal probes can often over-penetrate or damage tissue, especially when navigating the curved sulcus areas around implants. To mitigate these risks, studies suggest using a limited probing force of around 0.25 N, which helps ensure accurate measurements without compromising tissue integrity.

**Figure 13:**
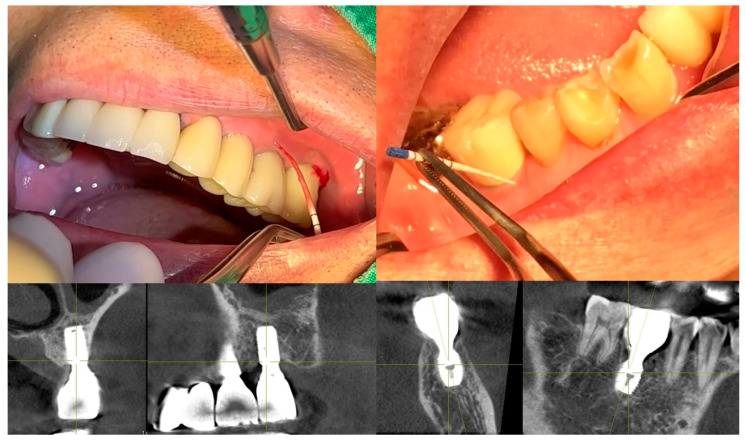
Implant paper point probing (IPPP) for evaluating peri-implant sulcus health. This figure illustrates the use of implant paper point probes (IPPPs), which have a yield strength of 0.25 to 0.35 N, for assessing sulcus depth around implants. The images compare peri-implant sulcus conditions in healthy and diseased implants. In cases of peri-implantitis, the paper points penetrate the sulcus without resistance and exhibit blood wetting upon withdrawal, indicating compromised soft tissue integrity. Conversely, in healthy peri-implant soft tissue, the paper points do not penetrate, despite the presence of a long soft tissue zone, confirming effective soft tissue sealing and biological stability.

The use of implant paper point probes (IPPPs) proved effective in evaluating the sealing capacity of peri-implant soft tissues:

- Controlled Force Application: With a yield strength of 0.25–0.35 N, the IPPP (Sure Dent Corp., Seoul, Republic of Korea) bends under excessive pressure, preventing tissue damage and allowing for accurate penetration (Figure 13)
- Reliable Results: The findings showed that SPI cases demonstrated intact sealing ability, highlighting the role of resilient connective tissue in maintaining peri-implant health.

### 9.4. Advantages of the IPPP Technique

1. Controlled Force: Minimizes the risk of over-penetration and tissue damage, offering a safer alternative to conventional metal probes.
2. Dual Diagnostic Functionality: Capable of detecting sulcus fluid and bleeding on probing (BOP), providing valuable diagnostic insights into tissue health.
3. Enhanced Adaptability: Unlike rigid metal probes, the IPPP conforms to the curved anatomy of the peri-implant sulcus, ensuring more reliable measurements.

This innovative approach confirmed the resilience and stability of peri-implant soft tissues in SPI cases, contributing to their long-term success and minimal bone changes.

### 9.5. Significance of IPPP in Peri-Implant Diagnostic Assessments

Although probing techniques are commonly used to evaluate sulcus health, the precise extent of junctional epithelium coverage in human peri-implant tissues remains uncertain. From this perspective, the IPPP provides a valuable alternative, offering a more accurate and minimally invasive method for assessing peri-implant soft tissue adaptation. By refining probing techniques, clinicians can gain deeper insights into peri-implant biology, leading to more predictable implant outcomes.

### 9.6. Clinical Comparison of Peri-Implant Sulcus Conditions (Figure 13)

Figure 13 illustrates the diagnostic application of IPPP, comparing two distinct peri-implant sulcus conditions based on penetration resistance:

**Peri-Implantitis Case:** The paper point easily penetrates the sulcus, indicating weakened soft tissue integrity. Upon withdrawal, the paper point is stained with blood, reflecting soft tissue breakdown, inflammation, and a compromised biological seal.

**Healthy Peri-Implant Soft Tissue:** Despite the presence of a long peri-implant soft tissue zone, the paper point does not penetrate, confirming an effective biological seal.

This finding reinforces the concept that long soft tissue zones do not necessarily indicate pathology as long as biologic width integrity and peri-implant health are preserved.

This figure underscores the clinical relevance of IPPP in differentiating between healthy peri-implant soft tissue and peri-implantitis, reinforcing its practical value as a peri-implant diagnostic tool.

While it is well acknowledged that the peri-implant soft tissue seal plays a crucial role in maintaining implant health [34], few schematic models have been developed to support and apply this fundamental biological knowledge. Beyond X-rays, there are limited methods for objectively assessing peri-implant soft tissue health. Implant paper point probing (IPPP) allows for direct measurement and validation of the peri-implant soft tissue seal, particularly in SPI cases, where the seemingly deep placement often raises clinical concerns. However, this clinically evident measuring tool can dispel such concerns by easily demonstrating outcomes, especially when combined with other clinical observations.

Achieving optimal implant outcomes centers on protecting the underlying bone, which is clinically manifested as a stable bone level. The key factor in this process is the peri-implant soft tissue, which plays a fundamental role in both implant protection and esthetics. This study focuses on the topography of peri-implant soft tissue in relation to implant placement depth, particularly in SPIs. The critical component responsible for implant and crestal bone protection is the soft tissue in contact with the submucosal portion of the abutment and restoration above the FAC. In non-SPI cases, this contact occurs entirely at the supracrestal level, where the peri-implant soft tissue exhibits both immunologic and mechanical properties. Traditionally, this area has been referred to as biologic width; however, in 2017, the World Workshop on the Classification of Periodontal and Peri-Implant Diseases and Conditions, co-sponsored by the American Academy of Periodontology (AAP) and the European Federation of Periodontology (EFP), recommended renaming it supracrestal tissue attachment (STA) to provide a more anatomically accurate description of the soft tissue attachment in both natural teeth and implants.

To further distinguish peri-implant soft tissue from the biologic width of natural teeth, this study introduces new terminology to describe specific components within this region. While these components collectively fall under the broader category of peri-implant soft tissue or biologic width of implants, contemporary consensus has advocated replacing biologic width with supracrestal tissue attachment (STA) or supracrestal insertion [35,36,37]. The STA encompasses the junctional epithelium and supracrestal connective tissue, maintaining the same dimensions previously attributed to biologic width.

Nonetheless, in SPI cases, peri-implant soft tissue comprises both supracrestal (TZ) and subcrestal (SZ) components, forming a functional entity distinct from conventional supracrestal attachment. Given this distinction, this study retains the term biologic width of implants, despite being considered outdated in some contexts, to more accurately reflect the functional and structural components described herein. However, where appropriate, biologic width and supracrestal tissue attachment are used interchangeably to align with the contemporary literature while preserving conceptual clarity in the proposed model.

Finally, although this study is based on theoretical foundations and clinical experience, further research is necessary to substantiate these findings. Future investigations should include histological studies to validate the proposed peri-implant soft tissue model, as well as long-term clinical reports to assess its practical application in diverse clinical settings [35,38].

## 10. Conclusions

The management of peri-implant soft tissue in subcrestally placed implants (SPIs) necessitates a paradigm shift from conventional biologic width (or STA) models to a more comprehensive three-dimensional framework. This study introduces the concepts of the transitional zone (TZ) and subcrestal zone (SZ) to account for both the vertical and horizontal components of peri-implant soft tissue adaptation, providing clinically relevant guidelines for optimizing emergence profile design and peri-implant stability.

By integrating mathematical modeling, CBCT-based radiographic assessment, and structured peri-implant soft tissue classification, this framework enhances the precision of implant placement and prosthetic planning. The identification of self-stained soft tissue (SSST)—comprising the TZ and SZ—redefines peri-implant soft tissue behavior, distinguishing a biologically stable interface from pathological pocket formation.

Future research should focus on validating this model through histological studies and long-term clinical trials, refining its application to diverse clinical scenarios. By adopting a three-dimensional analytical approach, clinicians can achieve biologically driven implant restorations that promote long-term peri-implant health, esthetic predictability, and functional success.

## Figures and Tables

**Table 1:** A table for calculating the vertical distance required given each emergence angle and horizontal distance difference, applicable to bone level implant designs with IPS, would be beneficial for understanding and implementing these measurements.

**Diameter of Crown in the Cervical Area**	**Diameter of FAC/Or Starting Point**	**Horizontal Distance** **Difference on Each Side**	**Emergence Angle**	**Vertical Distance**	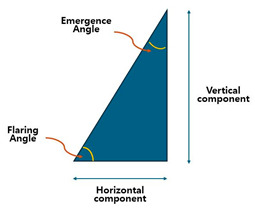
10	3	(10 − 3)/2 = 3.5	30°	6.1	
			45°	3.5	
			60°	2.0	
10	5	(10 − 5)/2 = 2.5	30°	4.3	
9	4		45°	2.5	
8	3		60°	1.4	

**Table 2:** Summary of peri-implant soft tissue parameters. This table defines the key parameters used to characterize peri-implant soft tissue structure and stability. It distinguishes measurable components (CRG, STT) from inferred components (CRL, SSST) and highlights their visibility in CBCT imaging. The table also illustrates how crestal restoration soft tissue (CRST) dynamically transitions into self-sustained soft tissue (SSST) when CRG is optimized, ensuring peri-implant health and stability. The reason why some parameters can be measured in CBCT while others cannot is due to the lack of a distinct demarcation between sulcular epithelium and junctional epithelium in radiographic imaging. This boundary can only be precisely identified through histological analysis, which is clinically impractical. However, indirect inferences can be drawn through clinical sulcus depth measurements or by assessing free-standing supracrestal tissue height (STH).

Parameter	Definition	Component Type	Visibility in X-Ray	Measurement/Inference
CRST (Crestal Restoration Soft Tissue)	Peri-implant soft tissue between the crestal bone and implant restoration.	Vertical and Horizontal	Partially visible (depends on thickness)	CRG (vertical) and CRL (horizontal) define its dimensions.
CRST (Crestal Restoration Soft Tissue)				
CRG (Crest to Restoration Gap)	Vertical distance from the crestal bone to the implant restoration, composed of void and soft tissue.	Vertical	Visible (depends on thickness)	Measured radiologically (CBCT). When optimized, CRG is converted into the STT of SSST (TZ and SZ).
CRL (Crest Restoration Length)	Horizontal dimension of the CRST.	Horizontal	Partially visible (e.g., palatal side)	Inferred based on CBCT and visible where tissue is thick enough (e.g., on the palatal side).
SSST (Self-Sustained Soft Tissue)	Optimized CRST, divided into the TZ and SZ, maintaining peri-implant tissue stability.	Vertical and Horizontal	TZ and SZ are inferred zones.	Vertical component measured with STT; horizontal component inferred using CRL.
SSST (Self-Sustained Soft Tissue)				
TZ (Transitional Zone)	Elastic soft tissue zone within the SSST, positioned coronally.	Vertical (STT) Horizontal (TZL)	STT visible	Vertical thickness measured via STT. TZL; inferred.
SZ (Subcrestal Zone)	Inelastic soft tissue zone within the SSST, located closer to the crestal bone.	Vertical (STT) Horizontal (SZL)	Visible and measurable	Vertical thickness measured via STT SZL; measurable.

## Data Availability

The data presented in this study are available within the article. No external datasets were generated or analyzed in this study.

## Abbreviations

SPI	Subcrestally Placed Implant
IPS	Internal connection with Platform Switching design
EA	Emergence Angle
FA	Flaring Angle
FA bl	Flaring Angle in the Buccolingual cross-section
FA md	Flaring Angle in the Mesiodistal cross-section
CRD	Crest to Restoration Distance
CRST	Crestal Restoration Soft Tissue
STT	Soft Tissue Thickness
TZ	Transitional Zone
TZL	Transitional Zone Length—the horizontal extent of the TZ
SZ	Subcrestal Zone
SZL	Subcrestal Zone Length—the horizontal extent of the SZ
SSST	Self-Sustained Soft Tissue
SSCT	Self-Sustained Connective Tissue
CBCT	Cone Beam Computed Tomography
IPPP	Implant Paper Point Probing
FAC	Fixture–Abutment Connection
BEMIR	Biologically Stable and Esthetic Molar Implant Restoration
BFP	Buccal First Point—the most coronal visible part of the implant restoration above the mucosa
MFP	Mesial First Point—the most coronal visible part of the implant restoration above the mucosa
DFP	Distal First Point—the most coronal visible part of the implant restoration above the mucosa
CP	Center Point—the reference point at the level of the edentulous ridge from which drilling starts
PCO	Peripheral Crestal Offset—the natural apical discrepancy between the central ridge and the peripheral crest
MDCS	Mesiodistal Crest Slope—the slope of the crestal ridge in the mesiodistal plane
CRG	Crest to Restoration Gap—the vertical distance between the crestal bone and the implant restoration
CRL	Crest to Restoration Length—the horizontal dimension of the Crest to Restoration Soft Tissue
STA	Supracrestal Tissue Attachment—the soft tissue structure consisting of the junctional epithelium and supracrestal connective tissue around natural teeth and implants.
